# Vapor of Volatile Oils from *Litsea cubeba* Seed Induces Apoptosis and Causes Cell Cycle Arrest in Lung Cancer Cells

**DOI:** 10.1371/journal.pone.0047014

**Published:** 2012-10-16

**Authors:** Soma Seal, Priyajit Chatterjee, Sushmita Bhattacharya, Durba Pal, Suman Dasgupta, Rakesh Kundu, Sandip Mukherjee, Shelley Bhattacharya, Mantu Bhuyan, Pranab R. Bhattacharyya, Gakul Baishya, Nabin C. Barua, Pranab K. Baruah, Paruchuri G. Rao, Samir Bhattacharya

**Affiliations:** 1 Centre for Advanced Studies in Zoology, School of Life Science, Visva-Bharati University, Santiniketan, West Bengal, India; 2 CSIR-North East Institute of Science and Technology, Jorhat, Assam, India; International Center for Genetic Engineering and Biotechnology, India

## Abstract

Non-small cell lung carcinoma (NSCLC) is a major killer in cancer related human death. Its therapeutic intervention requires superior efficient molecule(s) as it often becomes resistant to present chemotherapy options. Here we report that vapor of volatile oil compounds obtained from *Litsea cubeba* seeds killed human NSCLC cells, A549, through the induction of apoptosis and cell cycle arrest. Vapor generated from the combined oils (VCO) deactivated Akt, a key player in cancer cell survival and proliferation. Interestingly VCO dephosphorylated Akt at both Ser^473^ and Thr^308^; through the suppression of mTOR and pPDK1 respectively. As a consequence of this, diminished phosphorylation of Bad occurred along with the decreased Bcl-xL expression. This subsequently enhanced Bax levels permitting the release of mitochondrial cytochrome c into the cytosol which concomitantly activated caspase 9 and caspase 3 resulting apoptotic cell death. Impairment of Akt activation by VCO also deactivated Mdm2 that effected overexpression of p53 which in turn upregulated p21 expression. This causes enhanced p21 binding to cyclin D1 that halted G1 to S phase progression. Taken together, VCO produces two prong effects on lung cancer cells, it induces apoptosis and blocked cancer cell proliferation, both occurred due to the deactivation of Akt. In addition, it has another crucial advantage: VCO could be directly delivered to lung cancer tissue through inhalation.

## Introduction

Lung cancer is one of the most prevalent cancers and a major cause of worldwide cancer related death in approximately 1.4 million patients each year [1]. Among lung cancer, non small cell lung cancer (NSCLC) comprises ∼80% and within which adrenocarcinoma is considerably high in occurrence and mortality rate [2]. Chemotherapy and/or irradiation usually fails because NSCLC cells are intrinsically resistant to such therapies, moreover prognosis of NSCLC is notably poor [3], [4]. All these affected a very limited therapeutic choice for lung cancer. Hence there is a crucial need to develop a target specific chemo-intervention to retard cancer proliferation or to induce apoptosis or both to manage the problem of NSCLC.

To address the issue of NSCLC's alarming situation, several attempts have been made to search for suitable molecular targets to intervene cancer cells progression and apoptosis. In NSCLC cells, Akt/PKB is the constitutively active kinase which promotes cellular survival [5]. Activation of Akt occurs when it is recruited into the cell membrane through its PH domain and phosphorylated at Thr^308^ and Ser^473^ through the mediation of PDK1 (phosphoinositide dependent kinase 1) and mTOR (mammalian target of rapamycin) respectively [6], [7]. Interestingly, aberrant Akt activation greatly contributes to lung carcinogenesis [8]. Phosphorylated Akt (pAkt) is a powerful promoter of cell survival as it keeps this pathway alive by protecting Bcl-xL and antagonizing various components of the apoptotic cascades [9]. Although apoptotic response due to the inhibition of Akt has been observed at varying degrees in several types of cancers [10], [11] it could be crucial in lung cancer because enhanced phosphorylated form of Akt occurs perpetually [12].

Akt regulates p53, a tumor suppressor protein that controls cell cycle progression, through Mdm2 (murine double mutant-2), an ubiquitin ligase. Mdm2 is a substrate of Akt, phosphorylation of Mdm2 by Akt effects ubiquitination and proteasomal degradation of p53 [13]. Activated Akt therefore eliminates a major obstacle for cancer cell progression. Another important dimension of Akt is its inhibitory effect on apoptotic pathway. Bad, a member of Bcl_2_ family has been found to be the first protein that initiates apoptosis by displacing Bcl_2_ or Bcl-xL which allows Bax to oligomerize and create pores on the mitochondrial membrane to release cytochrome c into the cytosol [14], [15]. Activated Akt phosphorylates Bad at Ser136 causing it to dissociate from Bcl-xL from mitochondrial membrane and associate with an adaptor protein 14-3-3 resulting Bad sequestration to the cytosol [16], [17]. Target based amelioration from NSCLC cell progression or destruction is yet unavailable and since Akt is constitutively active here and the well characterized kinase known to support cancer cell survival and progression, its deactivation would be the best choice for dealing NSCLC.

In this report we demonstrate that volatile compounds from the oil extracted and purified from the seeds of *Litsea cubeba* (Lour.) Pers. (Lauraceae), a plant widely available in the North-East region of India, destroys lung cancer cells through the deactivation of Akt. Interestingly, it is the vapor of the oils which induces apoptosis and prevents cell proliferation of NSCLC by producing defects in Akt phosphorylation. The vapor of the oils demonstrated two prong effects, i.e., induction of apoptotic death and retardation of cell cycle progression, both occurs through the deactivation of Akt. This report therefore expected to have a special attraction as vapor induced destruction of lung cancer cells would have significant dimension in relation to its delivery to target tissue.

## Materials and Methods

### Reagents

All cell culture materials were obtained from Gibco-BRL, Life Technologies Inc., Gaithersburg, USA. The primary antibodies for pAkt (Thr308; sc-135650), pAkt1/2/3 (Ser473; sc-7985-R), Akt 1/2/3 (sc-8312), pPDK1 (Ser241; sc-101775), Bcl-xL (sc-7195), pBad (Ser136; sc-7999), Bad (sc-7869), pMdm2 (Ser166; sc-293105), p53 (sc-6243), p21 (sc-756), cyclin D1 (sc-753), poly[ADP-ribosyl]-polymerase (PARP) (sc-7150), Cytochrome c (sc- 7159) and β-actin (sc-130657) were purchased from Santa Cruz Biotechnology Inc., California, USA and mTOR (#2983) was procured from Cell Signaling Technology Inc., Danvers, MA, USA. Alkaline phosphatase and FITC conjugated secondary antibodies, Annexin V-Cy3 apoptosis detection kit, protein A agarose and CHAPS were purchased from Sigma Aldrich, St. Louis MO, USA. MTT assay kit was procured from Milipore, Temecula, CA, USA. Caspase-Glo™ 3/7 assay kit was acquired from Promega Corporation, Madison, WI, USA. Mitotracker was purchased from Molecular Probes, MD, USA, JC-1 mitochondrial membrane potential assay kit was purchased from Cayman Chemical Company (Ann Harbor, MI, USA). Apoptotic DNA ladder kit and BrdU labeling kit were procured from Roche Diagnostics (GmbH, Germany).

### Extraction and purification of essential oils from *Litsea cubeba* seeds

About 250 gm fresh ripe seeds of *Litsea cubeba*, collected during the months of August to October 2009–2011 from CSIR-NEIST experimental farm, Jorhat, Assam were soaked in distilled water and extracted using a Clavenger apparatus for 6 hours. The essential oil deposited above the water layer was separated using a separating funnel and dried over anhydrous sodium sulphate (neutral) and filtered to give oil (6.25 gm, 2.5% yield). The thin layer chromatography of the crude oil indicated the presence of four distinct spots. The crude oil (1 gm) was subjected to chromatographic purification in a silica gel (20 gm, 100–200 mesh, Rankem) column (1 inch diameter & 50 cm length) packed in hexane. 30 ml fractions were collected in the following order: fractions 1–10 (hexane), 11–20 (1% Ethyl acetate in hexane), 21–35 (2% Ethyl acetate in hexane), 36–60 (3% Ethyl acetate in hexane), and fraction 61-until completion of the elution of the compounds (4% Ethyl acetate in hexane). Fractions 11–20 containing 1 (henceforth referred as compound 1 or C1) (TLC) were combined and concentrated in a rotary evaporator to give an oil (100 mg) and this was identified as citronellal from comparison with authentic material (TLC, IR, NMR, MS). Fractions 23–35 containing 2 (henceforth referred as compound 2 or C2) (TLC) were combined and concentrated in a rotary evaporator to give an oily substance (86 mg) and was identified as neo-isopulegol by comparison of its ^1^H NMR spectrum with that reported in the literature [18]. Fractions 40–60 containing compound 3 (henceforth referred as compound 3 or C3) (TLC) were combined and concentrated in a rotary evaporator as explained earlier to give an oily residue (120 mg) and this was identified as isopulegol by direct comparison with ^1^H NMR spectrum with that reported in the literature [18]. Fractions 64–76 containing compound 4 (henceforth referred as compound 4 or C4) (TLC) were combined and concentrated to give a thick oil (55 mg) which was identified as citronellol from comparison of its ^1^H NMR spectrum with authentic sample.

Compound 1 (C1):

IR (CHCl_3_): υ 2925, 1724, 1457, 1437, 1219, 1040, 772 cm^−1^; ^1^H NMR (CDCl_3_, 300 MHz): δ 0.96 (d, *J* = 6.6 Hz, 3H, -CH*Me*), 1.30–138 (m, 2H, -CHMeC*H_2_*CH_2_-), 1.68 (s, 3H,  = C*Me*), 1.98 (s, 3H,  = C*Me*), 1.98–2.06 9m, 3H,  = C*CH_2_*- & -C*H*Me-), 2.24 (dd, *J* = 7.9, 2.6 Hz, 1H, -C*H*HCHO), 2.37 (dd, *J* = 5.4, 1.6 Hz, 1H, -CH*H*CHO), 5.06 (t, *J* = 7.0 Hz, 1H, -C*H* = CMe_2_), 9.75 (s, 1H, -C*H*O). MS (ESI): 155 (M^+^+1); bp 206°C (lit. 207°C).

Compound 2 (C2):

IR (CHCl_3_): υ 2925, 1722, 1643, 1455, 1445, 1375, 1219, 1024, 889, 772 cm^−1^; ^1^H NMR (CDCl_3_, 300 MHz): δ 0.87 (d, *J* = 6.6 Hz, 3H, -CH*Me*), 0.92–0.95 (m, 1H), 1.08–1.12 (t, *J* = 6.6 Hz, 1H), 1.47–1.54 (m, 1H), 1.68–1.75 (m, 3H), 1.79 (s, 3H, *Me*C = CH_2_), 1.95–1.99 (m, 2H), 3.98 (m, 1H, C*H*OH), 4.78 (s, 1H,  = C*H*_2_), 4.95 (s, 1H,  = C*H*_2_); MS (ESI): 154 (M^+^).

Compound 3 (C3):

IR (CHCl_3_): υ 2923, 1645, 1455, 1448, 1375, 1219, 1095, 1051, 1027, 886, 772 cm^−1^; ^1^H NMR (CDCl_3_, 300 MHz): δ 0.90–1.03 (m, 2H), 0.95 (d, *J* = 6.6 Hz, 3H, -CH*Me*), 1.30–1.35 (m, 1H), 1.47–1.54 (m, 1H), 1.63–1.65 (m, 1H), 1.69 (d, *J* = 1.5 Hz, 3H, *Me*C = CH_2_), 1.87–1.89 (m, 1H), 2.03–2.06 (m, 2H), 3.50 (dt, 1H, *J* = 10.4, 4.2 Hz, C*H*OH), 4.85 (s, 1H,  = C*H*_2_), 4.89 (s, 1H,  = C*H*_2_); MS (ESI): 154 (M^+^); bp 213°C (lit. 212°C ).

Compound 4 (C4):

IR (CHCl_3_): υ 3338, 2925, 1452, 1377, 1219, 1058, 1010, 738 cm^−1^; ^1^H NMR (CDCl_3_, 300 MHz): δ 0.91 (d, *J* = 6.6 Hz, 3H, -CH*Me*), 1.15–129 (m, 2H, -CHMeC*H_2_*CH_2_-), 1.33–1.45 (m, 2H, -C*H_2_*CH_2_OH), 1.51–1.53 (m, 1H, -C*H*Me-), 1.60 (s, 3H,  = C*Me*), 1.68 (s, 3H,  = C*Me*), 1.96–2.01 (m, 3H,  = C*CH_2_*- & O*H*), 3.61–3.74 (m, 2H, -C*H*_2_OH), 5.07 (t, *J* = 7.0 Hz, 1H, -C*H* = CMe_2_); MS (ESI): 157 (M^+^+1); bp 223°C (lit. 222°C).

### Cell culture and treatments

The lung cancer cell line A549, was a kind gift from Dr. Partha P. Banerjee (Georgetown University Medical Centre, Washington DC, USA), which he obtained from American Type Culture Collection (ATCC), USA. Cells were cultured in DMEM containing Earle's salts and non-essential amino acids supplemented with 10% fetal bovine serum, penicillin (100 U/ml) and streptomycin (100 µg/ml) in a humidified 95% O_2_/5% CO_2_ atmosphere at 37°C. Confluent cells were sub-cultured by trypsinization and subsequently seeded in 6 well culture plates containing DMEM with essential supplements.

Cells were seeded in a six well plate keeping one well devoid of cells. When confluency reached, crude oil or each individual compound or VCO were diluted in DMEM and applied in the empty well of the plate primed for treatment. VCO containing media was replenished every 24 h for the duration of the experiment. Cells were incubated at different time periods with several dilutions of VCO as mentioned under the figures. Control cells were kept in a separate incubator to avoid any exposure from VCO. At the end of the incubation, cells were lysed, centrifuged for 10 min at 10,000 g at 4°C and the supernatant was collected. Protein content of supernatant was determined by following the method of Lowry et al. [19]. Scrambled or p53 siRNA or Sp1 siRNA were transfected using Lipofectamine 2000 (Invitrogen, Carlsbad, CA, USA) following manufacturer's instructions.

### MTT cell viability assay

Cell viability was determined by using MTT assay kit (Milipore, Temecula, CA, USA) following manufacturer's instructions. Briefly, cells were plated in 96 well plates and were exposed to varied dilutions of crude oil or vapor oils for 72 h or VCO for different time periods. 10 µl of 3-(4,5-dimethylthiazol-2-yl)-2-5-diphenyl tetrazolium bromide (MTT) was added to each well for 4 h at 37°C. After solubilization in 100 µl 1(N) isopropanol/0.04(N) HCl, absorbance was read at 595 nm in a microplate reader (Thermo Electron Corporation, MA, USA).

### AnnexinV-Cy3 detection assay

AnnexinV-Cy3 apoptosis detection kit, purchased from Sigma Aldrich, St. Louis MO, USA was used to differentiate between live (green fluorescence), necrotic (red fluorescence) and apoptotic cells (green and red fluorescence). A549 cells incubated without or with VCO of dilution (2×10^3^) for 36 h were washed thoroughly with PBS, harvested and 50 µl of cell suspension was spotted on poly L-lysine coated glass slide. Cells were then washed thrice with binding buffer and stained with double labelling staining solution (Annexin V-Cy3 and 6 Carboxy Fluorescein Di-Acetate (CFDA) for 10 min. Excess labelling agent was removed by washing the cells three times with binding buffer and the cells were observed under fluorescence microscope (Zeiss Axio Scope A1, Carl Zeiss, Gottingen, Germany).

### JC-1 Mitochondrial Membrane Potential Assay

JC-1 staining was performed by using JC- 1 Mitochondrial Membrane Potential assay kit, Cayman Chemical Company, (Ann Arbor, MI, USA) as per the manufacturer's protocol. Briefly, control and VCO (2×10^3^ dilution) treated cells were subjected to JC-1 stain (10 µg/ml) for 20 min at 37°C. The shift of fluorescence due to VCO treatment was observed under fluorescence microscope (Zeiss Axio Scope A1, Carl Zeiss, Gottingen, Germany).

### DNA fragmentation assay

Low-molecular weight DNA was extracted from 1×10^6^ control or VCO (2×10^3^dilution) treated cells by using Apoptotic DNA ladder kit from Roche Diagnostics, (GmbH, Germany) following the manufacturer's instruction. Eluted DNA samples were then loaded on ethidium bromide stained 1.5% agarose gel and image was captured by Bio-Rad Gel Doc™ XR+, USA using Image Lab software.

### Electrophoresis and immunoblotting

60 µg of protein from control or treated cell lysates were resolved on 10% or 12.5% SDS-PAGE and transferred to PVDF membranes (Millipore, Bedford, MA) with the help of Semi-Dry trans blot Apparatus (TE77 Semi-Dry Transfer Unit, GE Healthcare, CA, USA). The membranes were first incubated overnight at 4°C with different primary antibodies at 1∶500 dilutions followed by respective alkaline phosphatase conjugated secondary antibodies at 1∶2000 dilutions at room temperature for 2 h. The protein bands were detected by using 5-bromro 4-chloro 3-indolyl phosphate/nitroblue tetrazolium (BCIP/NBT). Intensity of the bands was assessed by Image Lab software (Bio-Rad Gel Doc™ XR+, USA).

### Immunofluorescence study of cytochrome c

A549 cells were cultured on sterile uncoated glass cover slips. Both control and VCO (2×10^3^ dilution) treated cells were stained with Mitotracker (Molecular Probes, MD, USA) at 1∶10,000 dilutions (in DMEM) for 15 min. Cells were further washed with DMEM and processed for fixation. After fixation, cells were incubated with anti-cytochrome c antibody (1∶100) for 2 h followed by incubation with FITC-conjugated secondary antibody (1∶50) for another 1 h at room temperature. Cells were counter stained and mounted with DAPI mounting medium and observed under fluorescence microscope (Zeiss Axio Scope A1, Carl Zeiss, Gottingen, Germany).

### Caspase-Glo 3/7 assay

A549 cells were seeded in 96 well culture plates and incubated without or with VCO of dilution (2×10^3^) for different time periods. On termination of incubations, caspase activity was measured by using the Caspase-Glo™ 3/7, Promega Corporation, Madison, WI, USA according to the manufacturer's protocol. Luminescence was measured in a DTX-800 multimode detector (Beckman Coulter, CA, USA).

### BrdU incorporation assay

DNA synthesis was monitored by measuring incorporation of thymidine analogue 5-bromo-2′deoxyuridine (BrdU) in growing cancer cells using BrdU labeling and detection kit. Control and VCO (2×10^3^ dilution) treated A549 cells were refreshed with complete medium containing BrdU labeling reagent and incubated for 1 h. Cells were then washed thoroughly with wash buffer and fixed with ethanol fixative for 20 min at −20°C. Fixed cells were washed and incubated with anti-BrdU antibody solution followed by anti-mouse Ig fluorescein solution. Cells were then mounted on glass slides and observed under fluorescence microscope (Zeiss Axio Scope A1, Carl Zeiss, Gottingen, Germany).

### Chromatin immunoprecipitation (ChIP) assay

ChIP assay was performed by using a ChIP assay kit (Upstate, Temecula, CA, USA) following manufacturer's protocol using anti-p53 antibody. Primers were used for amplifying known p53 response element on p21 promoter and these are – RE1: forward: 5′-CAGGCTGTGGCTCTGATTGG-3′and reverse:5′-TTCAGAGTAACAGGCTAAGG -3′; RE2: forward: 5′-GGTCTGCTACTGTGTCCTCC-3′ and reverse: 5′-CATCTGAACAGAAATCCCAC-3′ [20] and PCR products were resolved on ethidium bromide stained 2% agarose gel and image was captured by Bio-Rad Gel Doc™ XR+, USA using Image Lab software.

### Co-immunoprecipitation (Co-IP) assay

200 µg of protein from control or VCO treated cells were incubated overnight with 2 µg of anti-p21 and anti-β-actin antibody at 4°C with continuous agitation. Protein A agarose was then added and incubated for 4 hours at 4°C. Antigen-antibody complexes were collected by centrifugation at 10,000 rpm for 2 minutes at room temperature. The pellet was washed 3 times to remove any unbound protein and boiled with 4× sample buffer for 5 min in a boiling water bath. The sample was centrifuged and the supernatant was loaded on 10%SDS-PAGE followed by immunoblotting with anti-cyclin D1 or anti-β-actin antibodies.

### Flow-cytometric Analysis

For cell cycle analysis, control and VCO (2×10^3^dilution) treated A549 cells were trypsinized, washed twice with PBS, and then fixed in 70% ethanol for 24 h before DNA analysis. After the removal of ethanol by centrifugation, cells were washed twice with PBS. Cells were then incubated with RNase (100 µg/ml) for 30 min and then stained with propidium iodide (PI-5 µg/ml) for 15 min. Fluorescence of the PI treated cells was measured with flow cytometer (BD FACSAria™ II). The percentages of G1, S, and G2-M cells were determined using the FACS Diva Software.

### Primary culture of normal lung cells

Normal lung cells were isolated from male Sprague Dawley rats following Phan et al. [21], briefly, normal healthy rats were anesthetised and the lungs were then perfused with sterile phosphate-buffered saline (PBS) through the right ventricle until they were pale. Then the lungs were removed, minced and digested in PBS containing 0.5% trypsin for 45 minutes at 37°C. Digested cells were separated from undigested tissue and debris by filtration through nylon mesh. Cells were then washed with PBS then with DMEM containing 10% fetal bovine serum and antibiotics, and finally resuspended in the medium. Cells were then plated in a six well plate keeping one well devoid of cells and incubated in a humidified 95% O_2_/5% CO_2_ atmosphere at 37°C. After 1 hour, cells were incubated with 2×10^3^ dilution of C2 or C3 or C4 or VCO for 12 hours. Control cells were kept in a separate incubator to avoid the presence of vapor. At the end of the incubation, cells were lysed, centrifuged for 10 min at 10,000 g at 4°C and the supernatant was collected. Protein content of supernatant was determined by following the method of Lowry et al. [19].

### Statistical analysis

Data were derived from at least three independent experiments and analyzed by one-way analysis of variance (ANOVA) where the F value indicated significance, means were compared by a post hoc multiple range test. All values were means ± SEM.

## Results

### Bioactivity guided isolation and purification of oils from the seed of *Litsea cubeba*

**Figure 1:**
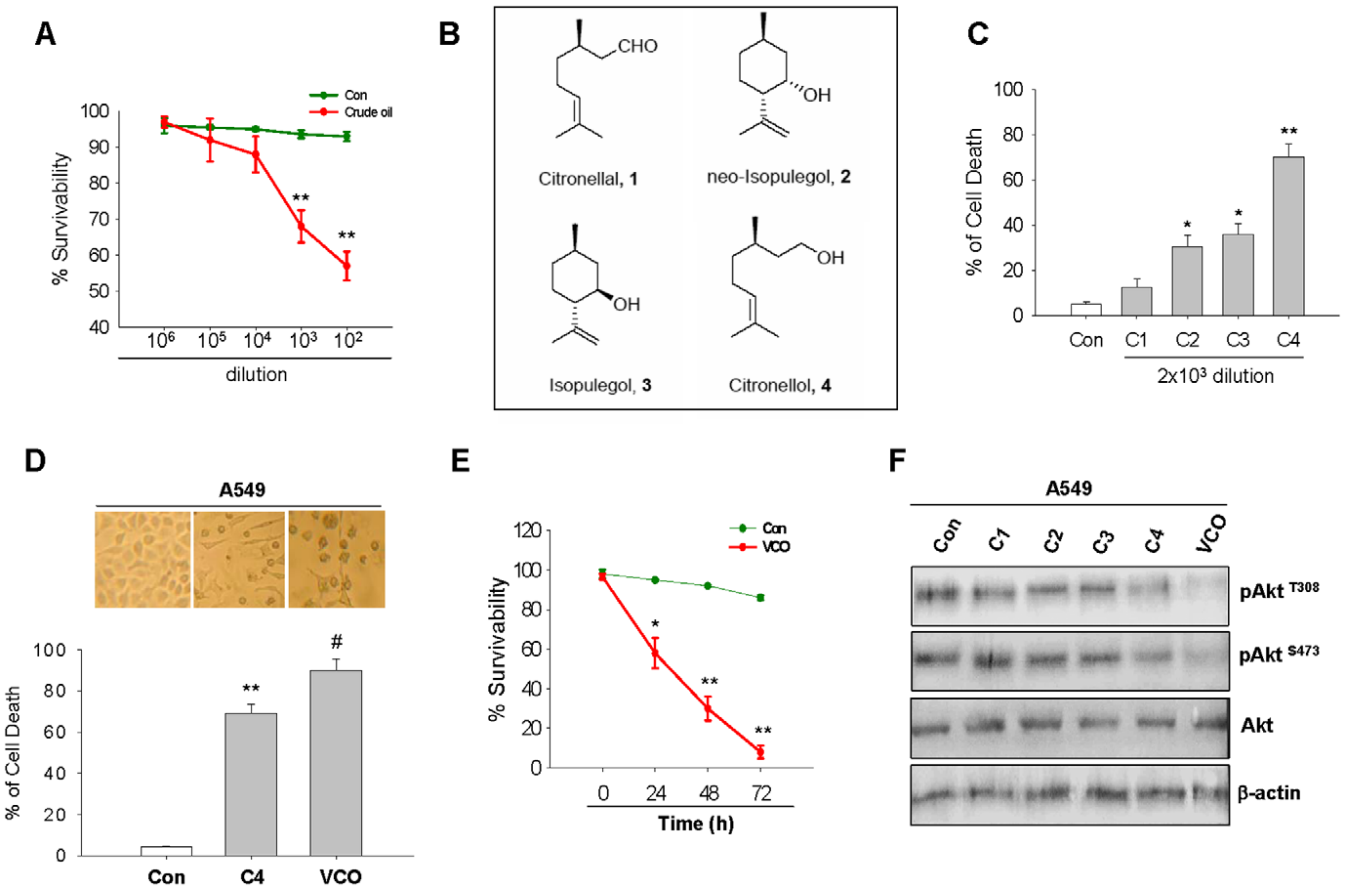
Effect of VCO on the viability of A549 cells by MTT assay. (**A**) Cell viability of A549 lung cancer cells were measured when exposed to vapors of different dilutions (10^6^ to 10^2^) of crude oil for 72 h by using MTT assay and the data was expressed as % of cell survivability relative to control. (**B**) Chemical structures of four most available compounds (C1- Citronellal; C2- neo-isopulegol; C3- isopulegol; C4- citronellol) isolated from *Litsea cubeba* seed essential oil. (**C**) Percentage of cell death was observed when A549 cells were exposed individually with these compounds for 72 h. (**D**) Effect of VCO (C2∶C3∶C4 as 1∶1∶1) and C4 on cell death at 72 h was observed by MTT assay, which was visualized by microscopic images. (**E**) Cell survivability was measured at different time intervals (24, 48, 72 h) with VCO exposure on A549 cells. (**F**) Western blot of Akt phosphorylation at Thr^308^, Ser^473^ and total Akt in A549 cells treated without (Con) with C1, C2, C3, C4 and VCO for 36 hours. β-actin served as internal loading control. Values are means ± SEM of 3 individual experiments. *p<0.05, **p<0.01 versus control and #p<0.05 versus C4.

The extracted crude oil vapor from *Litsea cubeba* seeds was examined for anti-cancer activity in A549 lung cancer cells. Several dilutions from crude oil were prepared and each dilution was added in one of the 6 well of culture plates while other 5 wells contained A549 NSCLC cells. The vapor generated from each dilution was expected to be spreading throughout the plate and reach cancer cells. Dose dependent exposure of vapor decreased the viability of A549 cells significantly at 72 h with [10^3^] and [10^2^] dilutions whereas until [10^4^] dilutions the vapor had no notable effect on survivability of cells (Fig. 1A). Chromatographic purification of *Litsea cubeba* seed essential oils gave rise to four types of compounds, identified through Mass and ^1^H NMR spectrum with authentic compounds and their chemical nature was detected as follows – C1: citronellal; C2: neo-isopulegol; C3: isopulegol and C4: citronellol (Fig. 1B), each was separately added at a dilution of [2×10^3^] in one of the 6 well culture plate, other 5 wells contained A549 cells. The vapors generated after addition of the oil to the well were exposed to cells for 72 h. It could be seen from Fig. 1C that C1 had poor activity, C2 and C3 exhibited 3-fold higher activity as compared to C1, while C4 had highest activity, so far cell mortality is concerned.

We combined C2, C3 and C4 at 1∶1∶1 ratio to observe whether the vapor out of these combinations could produce additional or synergistic effect on cell death over C4. The vapor from the combined oils (VCO) exhibited significantly (p<0.05) greater activity in killing A549 cells in comparison to the vapor of C4 alone (Fig. 1D) indicating that C2 and C3 produce additional effects to C4. We therefore used the vapor from these combined oils i.e. VCO. When VCO was exposed at different time periods, death of cells occurred linearly against time (Fig. 1E), indicating possibility of apoptosis induction by VCO. We have performed experiments to examine the potentiality of C1, C2, C3, C4 compound by determining inhibition of Akt activation. Since in A549 lung cancer cells pAkt is constitutively expressed, Akt phosphorylation was taken as marker to evaluate the anticancer effect. C4 showed greater inhibitory effect as compared to other compounds (C1, C2, C3,) but VCO which is a combination of C2, C3, and C4 produced significantly higher inhibitory effect when compared with C4 (Fig. 1F). At this point whether VCO produces cytotoxic effect on normal lung cells would be a relevant question. To observe this, vapor of C2, C3, C4 individually and VCO was exposed to primary culture of lung cells prepared from rat. Vapor from the oils and VCO did not produce any adverse effect in the cell morphology when compared with the untreated cells. In addition, we have also determined the inhibition of pAkt due to VCO in normal lung cells and found that there was no alteration in pAkt (Fig. S1A and B). All these suggest that at this dose of VCO, normal lungs cells are not affected whereas same dose effected considerable mortality of lung cancer cells.

### VCO induces apoptosis in lung cancer cells

**Figure 2:**
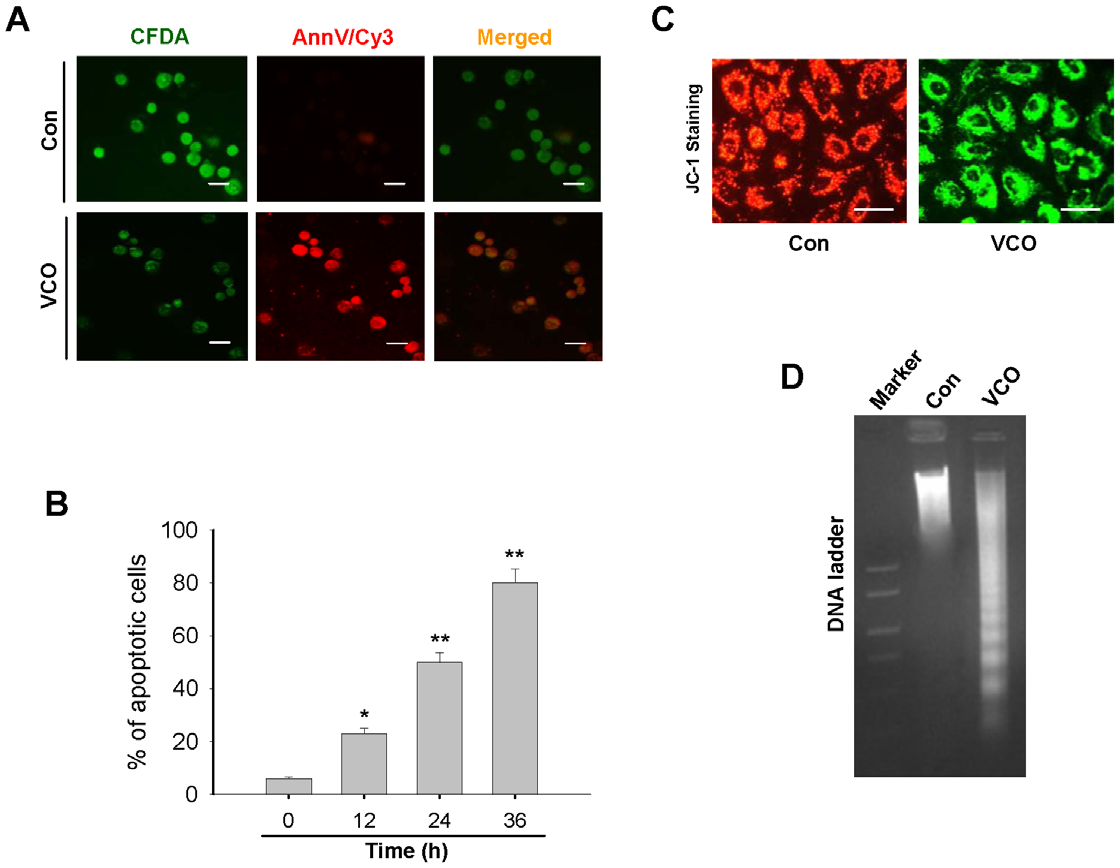
VCO induces apoptosis in A549 lung cancer cells. (**A**) Annexin-Cy3 (red) and 6-CFDA (green) double staining of apoptotic cells was examined by fluorescence microscopy where VCO treated A549 cells showed both green and red stains and control (untreated) cells stained green only. (**B**) Percentage of apoptotic A549 cells was measured at different time points (0 h, 12 h, 24 h, 36 h) with VCO treatments. (**C**) Mitochondrial membrane potential was observed in control and VCO exposed (36 h) A549 lung cancer cells by JC-1 staining assay. (**D**) Apoptotic DNA fragmentation was observed by VCO treated A-549 cells on 1.5% agarose gel electrophoresis. Data are presented as means ± SEM of three independent experiments. *p<0.05, **p<0.01 versus control (0 h). Bar represents 20 µm.

To examine the VCO effect on A549 lung cancer cell death, we used double fluorescence staining with 6-CFDA and Annexin V-Cy3 for differentiating the live, apoptotic, and necrotic cells. VCO-induced phosphatidylserine translocation from the inner to the outer leaflet of the plasma membrane was recognized by the phosphatidylserine-binding protein Annexin V conjugated with Cy3. At 36 h, control A549 cells showed staining only with 6-CFDA (green) whereas treatment with VCO increased the number of Annexin V-Cy3 (red) and 6-CFDA (green) double-stained cells (Fig. 2A) and this increased with time indicating enhancement of apoptotic cells (Fig. 2B). To extend our observation further, we used JC-1 fluorescent dye for examining mitochondrial membrane potential. In live cells, due to the physiological membrane potential, JC-1 associated with the mitochondrial membrane and form J-aggregates that emit red fluorescence while depolarized mitochondrial membrane in apoptotic cells contained monomeric JC-1 that fluoresce green. It could be seen from Fig. 2C that A549 cells were emitting red fluorescence whereas VCO incubated cells were marked with green fluorescence indicating cellular apoptosis. VCO induced apoptotic cell death in lung cancer cells was also evident from DNA ladder due to oligonucleosomal fragmentation of chromatin (Fig. 2D). These results indicate that VCO induces apoptosis only in lung cancer cells.

### Inhibition of Akt phosphorylation by VCO adversely affects down stream signaling for cell survival

**Figure 3:**
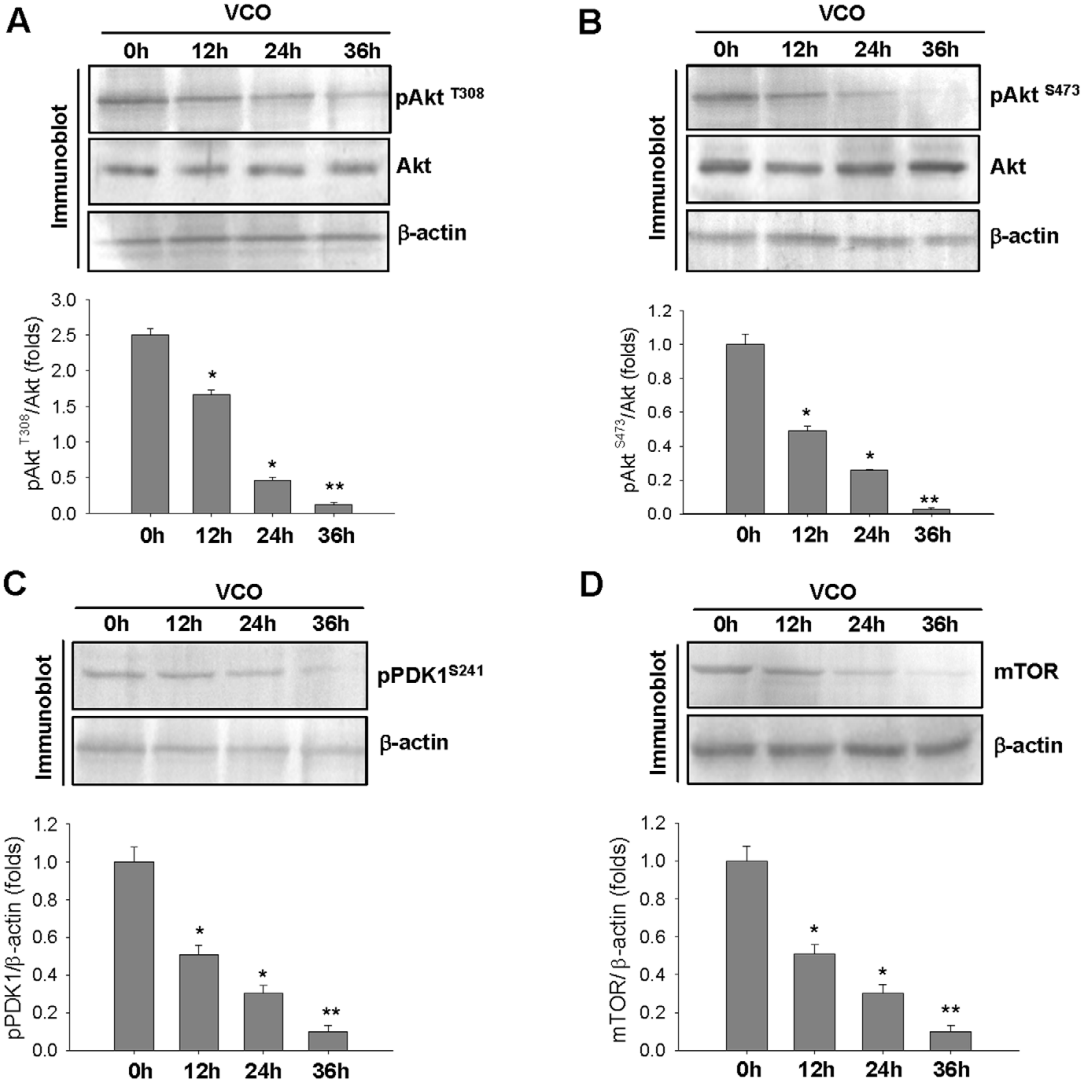
Time dependent inhibition of Akt phosphorylation by VCO. (**A**, **B**) Immunoblot analysis of Akt phosphorylation at Thr^308^ (A) and Ser^473^ (B) in A549 treated cells with VCO for the indicated time period (upper panel). Fold change represents the protein level of the VCO treated cells relative to the control cells. Bands were quantified by densitometric analysis where pAkt level was then normalized to the total Akt level (lower panel). β-actin served as loading control. (**C**, **D**) Immunoblot analysis of pPDK1 Ser ^241^ (C) and mTOR (D) was done at different time hour (0 h, 12 h, 24 h, 36 h) exposure of VCO to A549 cells (upper panel). Bands were quantified by densitometric analysis where pPDK1 or mTOR level was then normalized with β-actin which is represented by folds change (lower panel). Figures are representative of three independent experiments, *p<0.01, **p<0.001 versus control (0 h).

**Figure 4:**
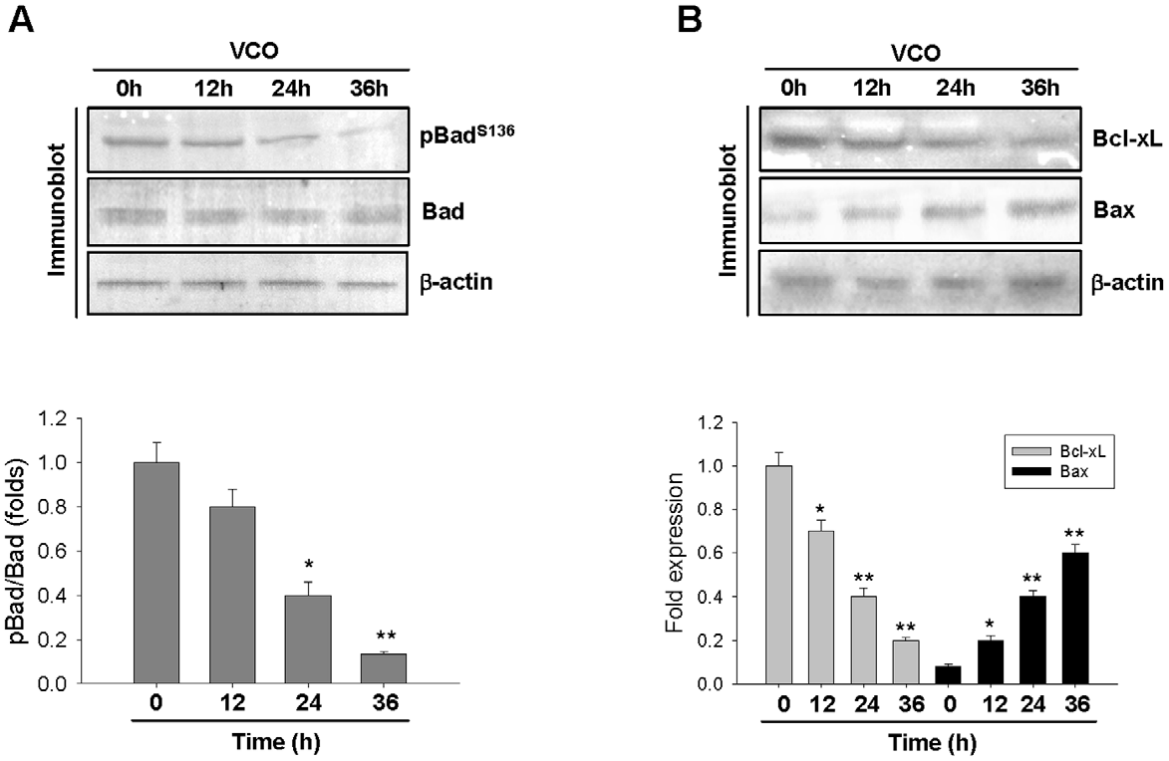
Deactivation of Bad with altered Bcl-xL/Bax ratio on mitochondrial membrane by VCO exposure. (**A**) Immunoblot analysis was performed to evaluate the level of pBad Ser^136^ and Bad in A549 cells exposed with VCO for different time periods (0 h, 12 h, 24 h, 36 h). β-actin served as internal control. Bands were quantified by densitometric analysis where pBad level was compared with Bad level. (**B**) Protein level of Bcl-xL and Bax of these cells were also evaluated by immunoblot analysis. Densitometric analysis showed Bcl-xL was negatively correlated with Bax level when A549 cells were exposed with VCO. Values are means ± SEM of three independent experiments, *p<0.05, **p<0.01 versus control (0 h).

In majority of cancer cells, Akt is a primary choice for the therapeutic intervention since it plays a key role in promoting immortality and proliferation of cancer cells. Phosphorylation of Akt at Thr^308^ and Ser^473^ are critical in maintaining these two vital characteristics. VCO treatment dramatically decreased Akt phosphorylation at Thr^308^ and Ser^473^ without any notable alteration of total Akt protein level in A549 cancer cells (Fig. 3A, B upper panel). 36 h of VCO treatment reduced pAkt Thr^308^ level by 70% and pAkt Ser^473^ level by 95% as compared to 0 h i.e. control cancer cells (Fig. 3A, B lower panel). This indicates that VCO strongly deactivates Akt which permits apoptotic pathway to progress. Activation of Akt is regulated by two discrete kinases– mTOR and PDK1, former is responsible for Akt phosphorylation on Ser^473^ while latter phosphorylates at Thr^308^. We therefore examined PDK1 phosphorylation and mTOR expression in response to VCO. There was significant decline of PDK1 phosphorylation and mTOR expression in A549 cells due to VCO exposure (Fig. 3C, D). Since Akt mediates its effect on the inhibition of apoptosis through Bad, deactivation of Akt due to VCO could seriously compromises with the activation of Bad. There was a significant decrease of Bad phosphorylation by VCO at 36 h which is an expected outcome of diminished Akt phosphorylation (Fig. 4A upper and lower panel). However, Bad protein in cancer cells remained unaltered during VCO treatment. Dephosphorylation of Bad results its translocation to the outer mitochondrial membrane that permits it to bind to anti-apoptotic protein Bcl_2_ family proteins, Bcl_2_ or Bcl-xL, which allows pro-apoptotic protein Bax to promote apoptosis. It could be seen from Fig 4B that subdued Bcl-xL level due to VCO at 24 h and 36 h, coincided with the decline of Bad phosphorylation. Decrease of Bcl-xL consequently resulted in the elevation of Bax protein (Fig. 4B upper and lower panel). Once Bcl-xL was displaced and Bad allows Bax to act, the event following this would be the release of cytochrome c from mitochondria.

### VCO exposure caused cell death through caspase pathway

**Figure 5:**
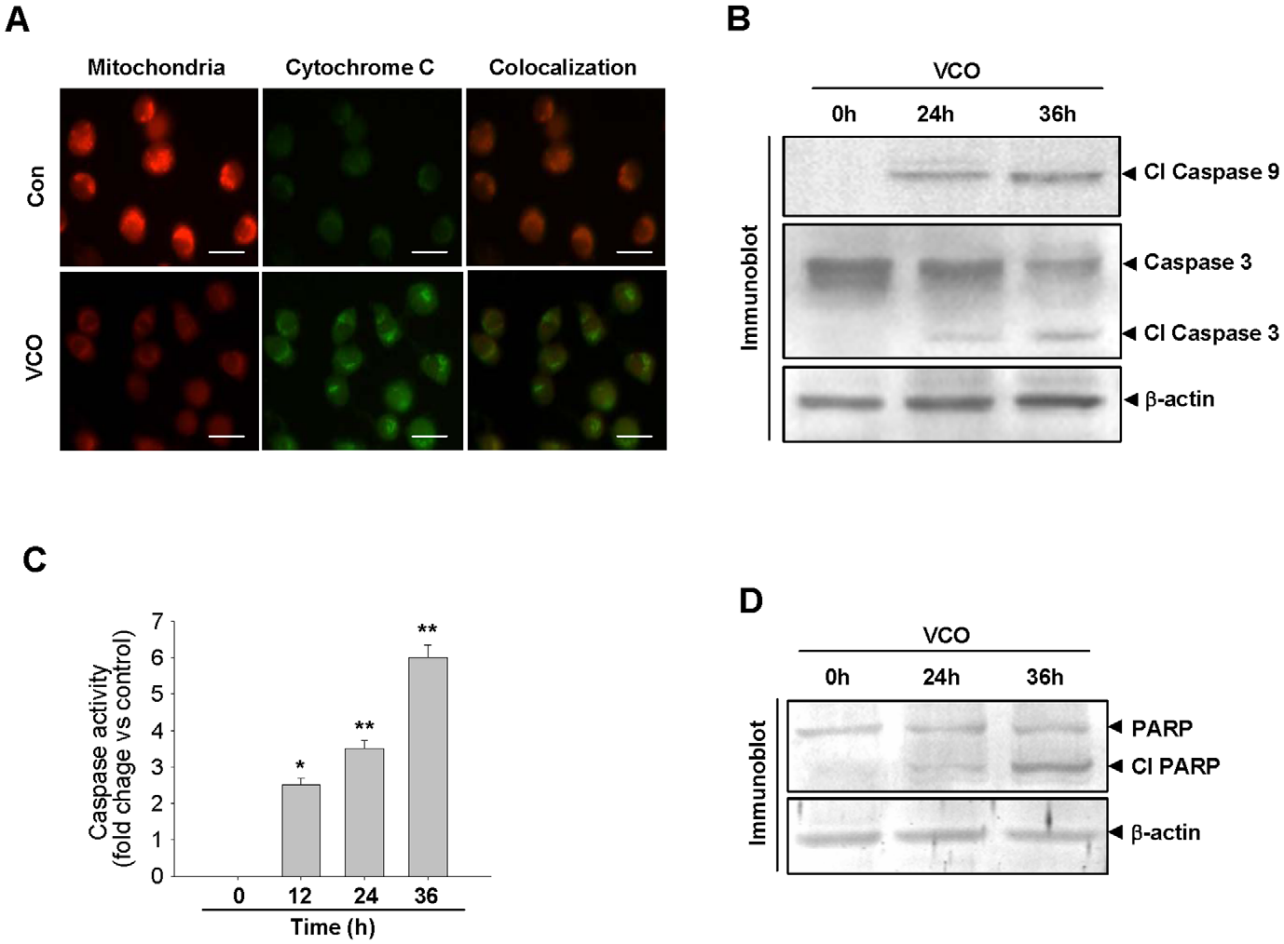
VCO induces apoptotic cell death by activating caspase cascade. (**A**) A549 cells were exposed with VCO for 36 h followed by staining of mitochondria with Mitotracker (red) and cytochrome c with FITC conjugated anti-cytochrome c antibody (green). (**B**) Immunoblot analysis was done by using anti-cleaved caspase-9 or caspase-3 antibodies in A-549 cells incubated in the presence of VCO at 0 h, 24 h, 36 h time intervals. β-actin used as internal control. (**C**) A549 cells were exposed with VCO for indicated time periods and on termination of exposure, cells were lysed and caspase 3 activity was measured in DTX multimode detector by using proluminescent caspase 3 as the substrate. (**D**) PARP cleavage was observed in VCO exposed cells by immunoblot analysis using anti-PARP antibody. β-actin used as loading control. Values are means ± SEM of three independent experiments, *p<0.01, **p<0.001 versus control (0 h). Bar represents 20 µm.

Since cytochrome c release from the mitochondria to the cytosol activates caspase pathway, we determined its release from the mitochondria in response to VCO by dual staining cytochrome c with FITC and mitochondria with mitotracker. VCO exposure notably released cytochrome c from mitochondria to cytosol (Fig. 5A), indicating possible initiation of apoptosis. Activation of caspases is the major event in apoptotic cell death. On receiving the death signal, inactive initiator caspase 9, which are present as zymogens, gets activated and cleaved, this cleaved product in turn activates the effector caspase 3 [22]. VCO treatment in A549 cells effected increase in cleaved caspase 9 formation which converted caspase 3 to cleaved caspase 3 (Fig. 5B). We analysed caspase 3 activity in response to VCO and found 6 fold increase of its activity in comparison to control cancer cells at 36 h (Fig. 5C). Time dependent increase of caspase 3 activity due to VCO was also reflected by the poly [ADP-ribosyl]-polymerase or PARP cleavage. PARP is a DNA repair enzyme, it is one of the substrates of caspase 3 and it would be evident from Fig 5D that PARP cleavage in A549 cells was substantially increased at 36 h at a time when caspase 3 activity was considerably high. This indicates irreparable damage of DNA, an event that occurs during apoptosis.

### Impairment of cyclin D1 by VCO

**Figure 6:**
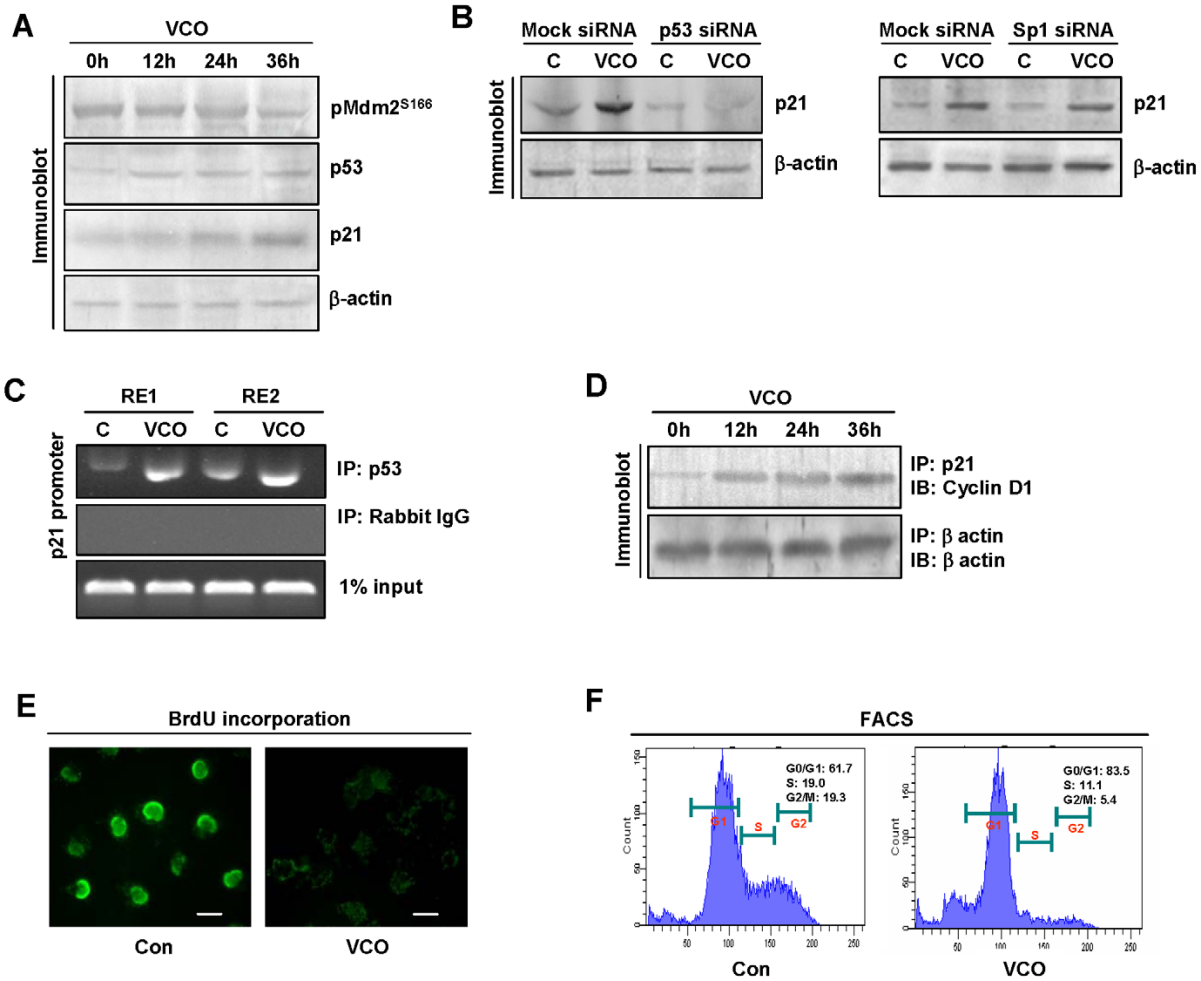
VCO halted cell cycle progression at G1- S phase by impairing Cyclin D1. (**A**) Immunoblot analysis of pMdm2 Ser^166^, p21 and p53 was analyzed in control or VCO exposed A549 cells at indicated time periods. β-actin used as internal loading control. (**B**) Immunoblot showed p21 level in p53 siRNA or Sp1 siRNA or their control siRNA transfected A549 cells exposed with or without VCO. (**C**) ChIP assay demonstrated VCO exposure increases binding of p53 to its response element (RE1 and RE2) on p21 promoter. (**D**) Cyclin D1-p21 interaction was increased with increasing the time of VCO exposure, which was shown by co-immunoprecipitation study. (**E**) BrdU incorporation in control and VCO treated A549 cells were examined by florescence microscopy. (**F**) FACS analysis showed cell cycle arrest at G1 to S phase as indicated by increased percentage of G_0_/G_1_ cells with the decrease of S and G_2_/M phase cells. Bar represents 20 µm.

Cyclin D1 is a key regulator of cell cycle progression, it is found to be overexpressed in lung adenocarcinoma which is related to its increased proliferation [23]. Augmented cyclin D1 activity for the enhancement of cell cycle progression in cancer cells could not occur in the presence of p53, because it enhances p21 expression and that in turn impairs cyclin D1-CDK4/6 complex required to allow cell cycle progression through G1 phase [24]. VCO exposure of A549 overexpressed p53 which consequently increased p21 protein expression. This seemed to be due to Mdm2 dephosphorylation which is expected as Mdm2 is a substrate of Akt (Fig. 6A). p21 gene transcription is regulated by both p53 and Sp1 [25], [26], therefore possibility remains that VCO could also be involved in augmenting p21 promoter activation. To examine this, we performed siRNA driven silencing of p53 and Sp1 gene expression in A549 cells followed by VCO incubations. Determination of p21 expression by western blot showed that suppression of p53 reduced VCO induced p21 expression while silencing of Sp1 had no effect on p21 overexpression that occured due to VCO (Fig. 6B left and right panel). Chromatin immunoprecipitation showed that exposure of VCO to A549 cells enhanced p53 binding to both of its response element RE1 and RE2 on p21 promoter (Fig. 6C). This suggests that VCO mediated Mdm2 deactivation causes p53 overexpression which in turn effects transactivation of p21 promoter resulting enhanced p21 expression. To observe whether this overexpressed p21 is associated with cyclin D1 for interfering its activity, we performed coimmunoprecipitation assay and found that immunoprecipitation of p21 followed by probing with anti-cyclin D1 antibody showed an increased association of cyclin D1-p21 from 12 h to 36 h due to VCO exposure (Fig. 6D). Taken these together, one would expect a regression in cell cycle progress. This would be evident from the suppression of BrdU incorporation in A549 cells at 36 h indicating a halt in DNA replication (Fig. 6E). FACS analysis depicts the result of cyclin D1 activity inhibition by VCO. VCO exposed A549 cells showed higher cell count, 83.5%, in G0/G1 phase while only 11.1% cells was in the S phase (Fig. 6F), indicating VCO induces cell cycle arrest at G1 to S phase

## Discussion

In this report we have described the unique anti-lung cancer property of vapor generated from volatile oil compounds (VCO) which were extracted and purified from *Litsea cubeba* seeds. In the North-East region of India, *Litsea cubeba* fruits and seeds are edible both as fruit and medicine to protect them from worm infection [27]. Some of the tribes and local people of Arunachal Pradesh in the North East Himalayan belt utilize *Litsea* not only as fruit and condiments in cooking but also for heart disease and stomach disorder [28]. These suggest *Litsea* fruits and seeds to be non-toxic. The oils from seeds are volatile in nature and that provided us the opportunity to examine it for anti-lung cancer activity. VCO induces apoptotic death of NSCLC and blocks their proliferation by impairing cyclin D1 activity, both occurred through the deactivation of Akt. Activation of Akt is crucial not only for cancer cells survival but also to prevent apoptosis. Hence, pAkt is a key regulator of cancer cell fate. Akt also permits cell cycle progression in several cancer cells including lung cancer and most importantly Akt is constitutively active here. Deactivation of Akt is therefore could be a vital step in dealing lung cancer problem. Cure from NSCLC is still a problem because they often develop resistance to available drugs [5], [29]. Moreover, precisely targeted intervention through apoptosis and/or inhibition of cancer progression in NSCLC is still a critical requirement. On this background, VCO appears to have certain interesting properties – (a) it can be directly delivered to lung tissue through inhalation; (b) it induces apoptotic death and (c) halts cell cycle progression. All these together would be expected to offer substantial strength in dealing lung cancer.

Constitutive activation of Akt in NSCLC has led this cancer cells to evade apoptosis. Akt phosphorylation at Ser^473^ is considered to be a major requirement for Akt activity, it is correlated with poor prognosis [30], [31], but this has been contradicted by some authors who nullify Ser^473^ phosphorylation to be of any significance [32]. More reliable participation is available from Thr^308^ phosphorylation [33] and it correlates with notable poor survival of NSCLC patients [34]. In this scenario VCO plays a safe role, it inhibits both Ser^473^ and Thr^308^ phosphorylation of Akt. From the time kinetic study it would be evident that inhibition of Akt phosphorylation by VCO is an earlier event which subsequently follows with the phosphorylation of other downstream members in the Akt signaling pathway.

Akt phosphorylates Bad at Ser^136^ that promotes its association with 14-3-3 protein which sequesters it in the cytosol [16], [17]. VCO induced deactivation of Akt reduced Ser136 phosphorylation of Bad thus allowing it to interact with pro-apoptotic Bcl_2_ family member Bax to aggregate on mitochondrial membrane resulting release of cytochrome c to the cytosol, which triggers the pathway for caspase cascade in NSCLC cells. Activation of Caspase 9 and 3 affects inactivation of key DNA repair protein PARP, therefore cleavage of cellular DNA by caspase activated DNase does not allow the opportunity for their repair [35]. We have observed VCO induced fragmented DNA of NSCLC cells which indicate irreversible apoptotic destruction of these cancer cells.

Interestingly, VCO interferes with another important front of NSCLC through the deactivation of Akt. It blocks cell cycle progression which will obstruct their prolific growth. Mdm2, a ubiquitin ligase, is another good substrate for Akt, Akt phosphorylates Mdm2 on Ser^166^ and that promotes p53 protein ubiquitination and degradation [13], [36]. p53 restricts cell cycle progression, it transactivates p21 promoter that enhances p21 expression which then associates itself with cyclin D1 and retards its interaction with CDK4/6. This causes inhibition of G1 to S phase cell cycle progression [37]. We have observed that VCO reduced Mdm2 Ser^166^ phosphorylation, the site which Akt phosphorylates and this decrease permits p53 overexpression which in turn upregulates p21 that follows its greater association with cyclin D1. This creates an obstacle at G1 to S phase progression in these cancer cells.

In lung cancer, especially in NSCLC, there is practically very little option for chemotherapy. Cisplatin, paclitaxel, etoposides are of common choice [38], [39]. One of the major problems with these compounds is their toxicity [40], [41]. Since these chemotherapies are not very effective and have considerable side effects, attempts are recently made for target modulation therapies and combination therapies. Cisplatin based adjuvant chemotherapy appears to be better advantage in relation to the survivability [42]. Moreover Akt is known to play a crucial role in PI3k/Akt/mTOR pathway which is important in other types of cancers where numbers of inhibitors are examined in this direction [43]. Although Akt is an attractive target in global cancer, available drugs for managing lung cancer are not capable to target Akt in their mode of action. However, it is still difficult task to manage NSCLC by chemotherapy. On this background our present investigation with VCO provides encouraging possibilities as it targets Akt, a key player for the maintenance of survivability and propagation of lung cancer.

Immortality and prolific growth are two vital characteristics of cancer cells that prevent therapeutic intervention. Death of cancer cells through apoptosis sometime is not enough to treat the cancer especially when the rate of cell proliferation surpasses that of killing. This will permit the malignant growth of the tumor to persist and ultimately surrender to it. In contrast, when both growth and immortality are impaired, it would halt cancer progression and promise cure. VCO demonstrates this opportunity for lung cancer treatment, more importantly it is NSCLC which is the most prevalent adenocarcinoma and considerably fearsome as chemotherapy often fails in its treatment. VCO has another crucial advantage, being a vapor it may be delivered to lung tissue directly through inhalation. To our knowledge, no drug has yet been formulated for inhalation therapy in the case of lung cancer which obviously will minimize side effects, quantity to be delivered is expected to be low and this will be target specific.

## Supporting Information

Figure S1 **Effect of VCO on rat primary lung cells.** (**A**) Effect of three different compounds C2, C3, C4 and VCO exposed to primary culture of lung cells obtained from rat. (**B**) Immunoblot analysis of Akt phosphorylation at Ser^473^, Thr^308^ and Akt protein from lung cells treated with or without VCO. β-actin served as internal loading control.
(TIF)
