# Supplementary figures and images for: Vapor of Volatile Oils from Litsea cubeba Seed Induces Apoptosis and Causes Cell Cycle Arrest in Lung Cancer Cells
Source: PLoS One. 2012 Oct 16;7(10):e47014. doi: 10.1371/journal.pone.0047014 (PMC3473030; doi:10.1371/journal.pone.0047014)

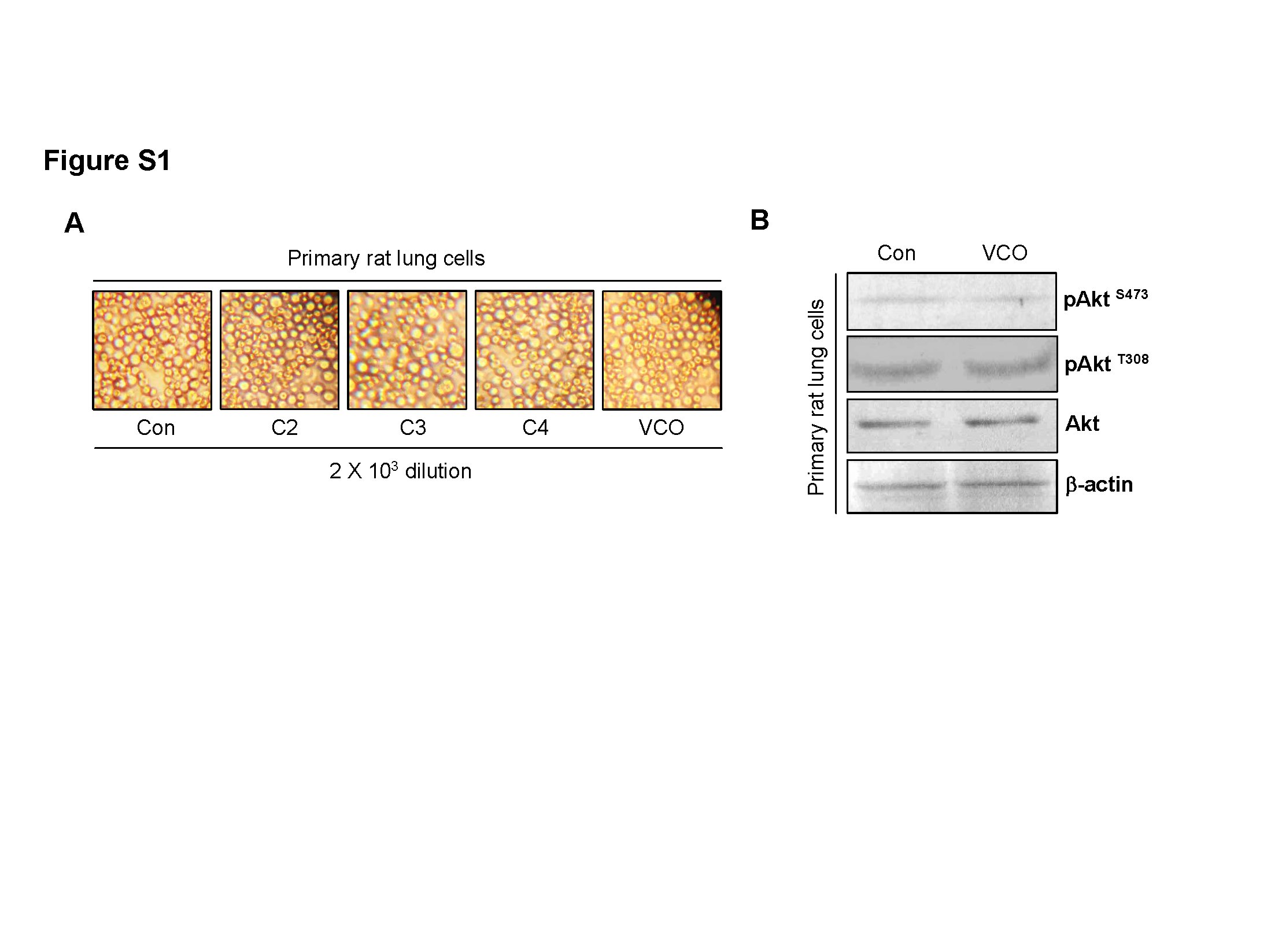

Supplement: Figure S1 — Effect of VCO on rat primary lung cells. (A) Effect of three different compounds C2, C3, C4 and VCO exposed to primary culture of lung cells obtained from rat. (B) Immunoblot analysis of Akt phosphorylation at Ser473, Thr308 and Akt protein from lung cells treated with or without VCO. β-actin served as internal loading control. (TIF) [file pone.0047014.s001.tif]
